# Time-Dependent Impact of Betulin and Its Derivatives on IL-8 Expression in Colorectal Cancer Cells with Molecular Docking Studies

**DOI:** 10.3390/ijms26136186

**Published:** 2025-06-27

**Authors:** Marcel Madej, Adrianna Halama, Elwira Chrobak, Joanna Magdalena Gola

**Affiliations:** 1 Department of Molecular Biology, Faculty of Pharmaceutical Sciences in Sosnowiec, Medical University of Silesia, 40-055 Katowice, Poland; jgola@sum.edu.pl; 2 Silesia LabMed, Centre for Research and Implementation, Medical University of Silesia, 40-752 Katowice, Poland; adrianna.halama@sum.edu.pl; 3 Department of Organic Chemistry, Faculty of Pharmaceutical Sciences in Sosnowiec, Medical University of Silesia, 40-055 Katowice, Poland; echrobak@sum.edu.pl

**Keywords:** colorectal cancer cell lines, interleukin, CXCL8, betulin, derivatives, mRNA, protein, molecular docking

## Abstract

Colorectal cancer (CRC) remains one of the most prevalent malignancies of the gastrointestinal tract worldwide, with chronic inflammation recognized as a key factor in its progression. Among pro-inflammatory cytokines, interleukin 8 (IL-8) plays a pivotal role in promoting angiogenesis, tumor cell migration, and metastasis. Elevated IL-8 expression is frequently associated with advanced CRC stages. This study investigated the effects of betulin and its semi-synthetic derivatives, EB5 and ECH147, on IL-8 expression in CRC cell lines characterized by differing malignancy grades. IL-8 transcript and protein levels were quantified using real-time RT-qPCR and a proximity ligation assay, respectively, following compound exposure at 2, 8, and 24 h. Basal IL-8 levels were significantly higher in low-grade CRC cell lines. Among the compounds tested, ECH147 exerted the most pronounced, time-dependent inhibitory effect on CXCL8 expression. Furthermore, molecular docking analyses revealed that ECH147 exhibits stronger binding affinity toward the IL-8 protein compared to conventional chemotherapeutics. These findings suggest that the modification of the betulin structure via the incorporation of a propynoyl moiety enhances both its molecular interaction with CXCL8 and its anti-inflammatory potential. ECH147 and EB5 thus emerge as promising candidates for further development as immunomodulatory agents targeting the IL-8-associated pathway in CRC.

## 1. Introduction

Gastrointestinal (GI) cancers represent a diverse group of malignancies with significant clinical impact. Colorectal cancer (CRC), in particular, ranks among the most prevalent and deadly tumors of the GI tract worldwide [1,2]. Its development is strongly associated with various risk factors, most of which are related to unhealthy lifestyle habits, such as physical inactivity, tobacco use, and diets that include highly processed foods. Only a small proportion of CRC cases are attributed to hereditary or genetic predispositions [1,3].

The diagnostic process for CRC is relatively accurate and primarily relies on endoscopic procedures, such as sigmoidoscopy and colonoscopy, which are often complemented by laboratory testing [1,2,3]. Once malignant lesions are detected, therapeutic options become more restricted. In the majority of cases, treatment involves the surgical resection of the affected section of the colon, typically followed by chemotherapy. Although this approach is generally effective, it can significantly impact the patient’s quality of life and may also damage healthy tissues [1,2,3].

The most commonly used chemotherapeutic agents include 5-fluorouracil (5-FU) and its prodrug capecitabine, which is metabolized into 5-FU in vivo. Combination chemotherapy regimens are widely employed, such as FOLFOX-4 (which includes oxaliplatin—a platinum-based alkylating agent—administered with 5-FU and leucovorin) and FOLFIRI (irinotecan in combination with 5-FU and leucovorin) [3,4]. In selected patients, targeted therapies are also applied, particularly immunotherapies that involve monoclonal antibodies [3,4]. A prominent example is cetuximab (IgG1), which binds to the epidermal growth factor receptor (EGFR), thereby interrupting intracellular signaling pathways and inhibiting tumor cell proliferation [3,4,5]. However, this therapy is only effective in patients with wild-type *KRAS* and *NRAS* genes, making prior molecular diagnostics essential. Additionally, clinical evidence suggests the reduced efficacy of cetuximab in patients who harbor the V-raf murine sarcoma viral oncogene homolog B (*BRAF*) mutation, which involves the conversion of valine (V) at locus 600 to glutamic acid (E) (*BRAF^V600E^*) and is associated with a poorer prognosis and limited response to anti-EGFR therapy [5]. Due to the considerable burden and cytotoxicity of current chemotherapy, which adversely affects healthy cells, there is a growing interest in alternative pharmacological strategies. Natural compounds have emerged as promising candidates, with particular attention given to betulin.

Betulin (lup-20(29)-ene-3β,28-diol), a pentacyclic triterpenoid of the lupane type, exhibits a wide range of biological activities, including anti-inflammatory, antibacterial, and potential anticancer effects [6,7]. This compound is primarily extracted from the outer bark of birch trees that belong to the Betula species. However, the application of betulin is limited by its relatively poor bioavailability, which is largely attributed to its chemical structure. To overcome this limitation, various chemical modifications have been explored to synthesize novel derivatives with improved pharmacokinetic and pharmacological properties [6,7].

In cancer-related diseases, increasing attention is being focused on the pro-inflammatory interleukin-8 (IL-8), which is more frequently referred to in the literature as chemokine (C-X-C motif) ligand 8 (CXCL8) [8,9]. IL-8 belongs to the chemokine family, which includes specific proteins with chemotactic properties that induce the forced migration of cells, mainly neutrophils, that express specific receptors to the site of inflammation, such as cancer cells [6,9]. It is primarily produced by activated macrophages, fibroblasts, cancer-associated fibroblasts (CAFs), and cancer cells [8,10]. CXCL8 can act as a monomer or dimer through its two G protein-coupled receptors. During prolonged inflammation in the body, IL-8 is secreted by these cells. Interaction with the CXCR1/CXCR2 receptor activates three molecular pathways: PI3K/Akt, the mitogen-activated protein kinase (MAPK) cascade, and FAK/Src, which confer chemoresistance to cancer cells [8,9,10]. The PI3K/Akt pathway is the main cascade triggered by IL-8, which leads to cancer cell survival, increased proliferation and migration, and the formation of new blood vessels [8,9,10]. Uncontrolled cell growth is ensured by the activation of the MAPK kinase pathway [8,9,10]. IL-8 also plays a crucial role in the tumor microenvironment, mainly through its paracrine and autocrine abilities, significantly affecting tumor-associated macrophages (TAMs) and CAFs. Through the paracrine action of this chemokine, other macrophages are activated, leading to the translocation of extracellular matrix metalloproteinases (MMPs) and EGFR, which are factors essential for the invasiveness and metastasis of cancer cells to distant regions, primarily the liver [8,11]. High levels of IL-8 are primarily observed in patients with advanced stages of cancer, as this chemokine is critically important for the migration and invasiveness of colorectal cancer cells [11].

Chronic inflammation is a key feature in the pathogenesis of CRC and is commonly associated with the increased expression of IL-8 [2,8]. Given that this cytokine plays a key role in CRC by promoting inflammation, enhancing tumor cell survival, and contributing to resistance to therapy, it is an attractive target for anticancer treatment. Natural compounds, such as betulin, have demonstrated anticancer activity; however, their chemical structure results in poor solubility and low bioavailability, which limits their therapeutic potential. To overcome these limitations, chemical modifications are introduced to improve their pharmacokinetic properties. The incorporation of functional groups, such as propargyl or phosphonate, into the betulin scaffold may not only enhance its bioavailability but also increase its ability to modulate IL-8 expression.

Therefore, this study investigates whether betulin and its synthetic derivatives, EB5 and ECH147, can differentially regulate IL-8 expression at both the transcriptional and translational levels in CRC cell lines that represent various stages of disease progression. This study aimed to assess the impact of betulin and its two synthetic derivatives, EB5 (28-propynoylbetulin) and ECH147 (29-diethoxyphosphoryl-28-propynoylbetulin), on CXCL8 expression at both the mRNA and protein levels in CRC cell lines SW1116, HT-29, and RKO. Normal epithelial cells (CCD-841CoN) were also included to assess the general impact of the compounds on non-cancerous cells. To further determine the pharmacological potential of these agents, their time-dependent effects on IL-8 expression and secretion were analyzed with the aim of identifying compounds that exhibit selectivity toward specific CRC cell lines that represent various stages of tumor progression. Additionally, molecular docking studies were conducted to explore the possible mechanism of action by visualizing compound–protein interactions involved in the modulation of IL-8 expression.

## 2. Results

### 2.1. Temporal Profiling of IL-8 Expression in Colorectal Cancer Cell Lines Treated with Investigated Compounds

**Figure 1:**
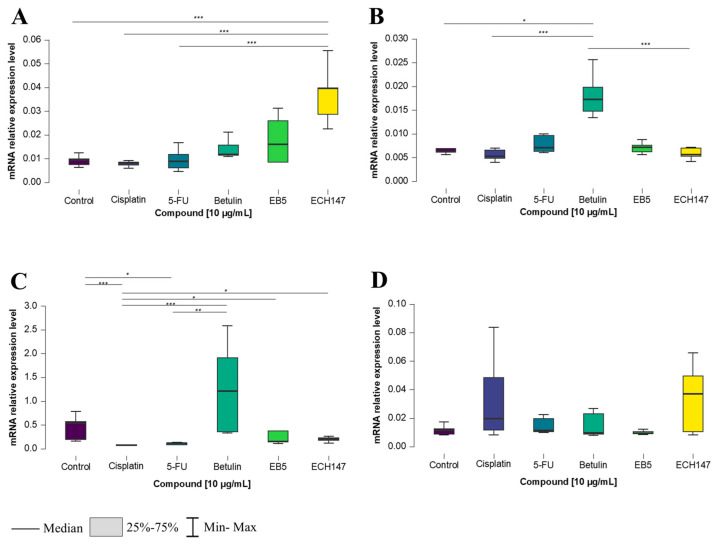
Changes in gene expression of *IL-8* in colorectal cancer cell lines after 2 h of exposure to the tested compounds: CCD-841CoN (**A**), SW1116 (**B**), HT-29 (**C**), RKO cell line (**D**). 5-FU—5-fluorouracil; EB5—28-propynoylbetulin; ECH147—29-diethoxyphosphoryl-28-propynoylbetulin; *—*p* < 0.05; **—*p* < 0.01; ***—*p* < 0.001.

To investigate the time-dependent effects of compound exposure at a concentration of 10 μg/mL on CRC cells, *IL-8* gene expression levels were measured after 2, 8, and 24 h in various colorectal cancer cell lines, as well as in a normal colonocyte line (CCD-841CoN). In the CCD-841CoN cell line (Figure 1A), a statistically significant increase in *IL-8* expression was observed 2 h after treatment with the ECH147 derivative, compared to untreated cells and reference compounds (cisplatin and 5-FU) (*p* < 0.001).

For the SW1116 cell line, the greatest upregulation of *IL-8* mRNA expression occurred following treatment with the unmodified triterpene (betulin), compared to untreated cells (*p* < 0.05), as well as in comparison to both cisplatin and ECH147 (*p* < 0.001) (Figure 1B).

A similar trend was observed in the HT-29 cell line, in which *IL-8* expression significantly increased after betulin treatment, compared to 5-FU (*p* < 0.01) and cisplatin (*p* < 0.001). Interestingly, a statistically significant increase in *IL-8* expression was also noted after treatment with ECH147, compared to cisplatin (*p* < 0.05), as well as EB5, compared to cisplatin (*p* < 0.05). Moreover, a decrease in *IL-8* mRNA expression was detected following treatment with 5-FU (vs. control, *p* < 0.05) and cisplatin (vs. control, *p* < 0.001) (Figure 1C). In contrast, the RKO cell line showed no statistically significant differences in *IL-8* gene expression after treatment with any of the tested compounds (Figure 1D).

**Figure 2:**
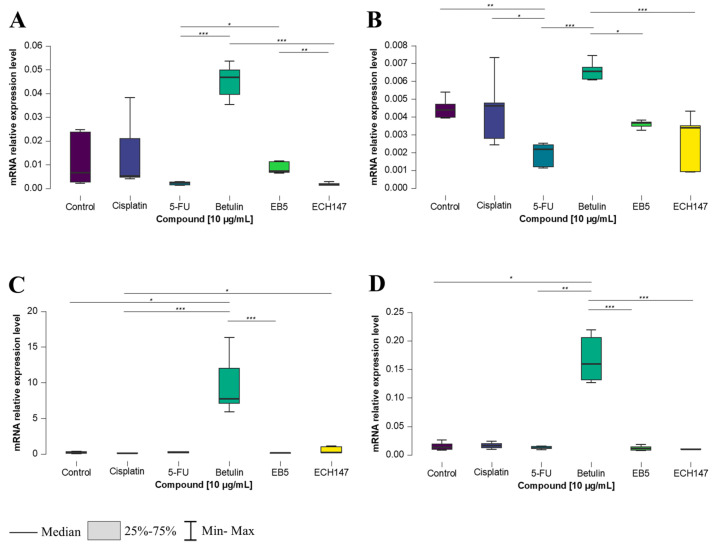
Changes in gene expression of *IL-8* in colorectal cancer cell lines after 8 h of exposure to the tested compounds: CCD-841CoN (**A**), SW1116 (**B**), HT-29 (**C**), RKO cell line (**D**). 5-FU—5-fluorouracil; EB5—28-propynoylbetulin; ECH147—29-diethoxyphosphoryl-28-propynoylbetulin; *—*p* < 0.05; **—*p* < 0.01; ***—*p* < 0.001.

After 8 h of exposure to the tested compounds, statistically significant changes in *IL-8* gene expression were observed in all examined CRC cell lines. Notably, in each of them, the greatest increase in *IL-8* expression was consistently observed following treatment with betulin. In the normal colon epithelial cell line, CCD-841CoN, betulin induced a statistically significant increase in *IL-8* expression compared to both EB5 and 5-FU (*p* < 0.001). Furthermore, a significant decrease in *IL-8* expression was observed after treatment with the ECH147 derivative, compared to EB5 (*p* < 0.01), and following 5-FU treatment, compared to EB5 (*p* < 0.05) (Figure 2A).

In the SW1116 cell line (Figure 2B), 8 h of exposure to the compounds led to a significant downregulation of *IL-8* mRNA expression after treatment with 5-FU compared to the control (*p* < 0.01), cisplatin (*p* < 0.05), and betulin (*p* < 0.001). Conversely, a significant increase in *IL-8* expression was observed after betulin treatment compared to EB5 (*p* < 0.05) and ECH147 (*p* < 0.001).

A similar effect of betulin on *IL-8* expression was observed in the HT-29 cell line (Figure 2C). A statistically significant increase in gene expression was found after treatment with the unmodified pentacyclic triterpene (betulin) compared to EB5 and 5-FU (*p* < 0.001), as well as compared to untreated cells (*p* < 0.05). Interestingly, a significant increase was also observed after treatment with ECH147 vs. the control (*p* < 0.05).

In the RKO cell line, *IL-8* expression levels after 8 h of compound exposure followed a pattern similar to that of the SW1116 cell line. A marked increase in *IL-8* expression was noted after treatment with betulin, compared to its derivatives, EB and ECH147 (*p* < 0.001), as well as in comparison with 5-FU and the untreated control (*p* < 0.01 and *p* < 0.05, respectively) (Figure 2D).

**Figure 3:**
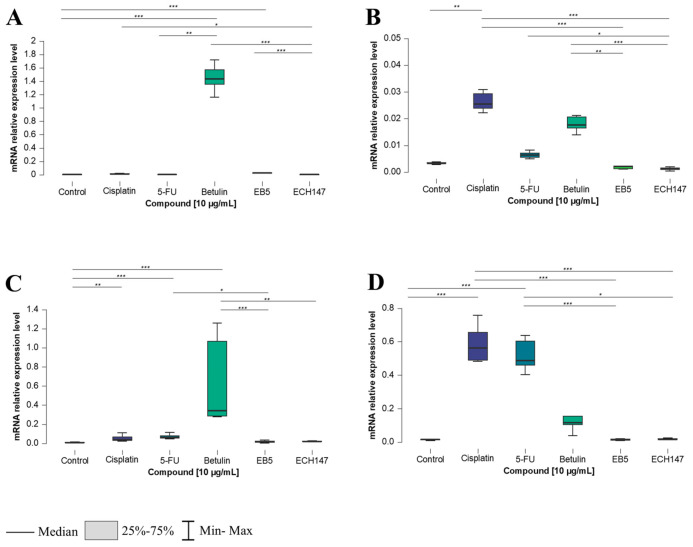
Changes in gene expression of *IL-8* in colorectal cancer cell lines after 24 h of exposure to the tested compounds: CCD-841CoN (**A**), SW1116 (**B**), HT-29 (**C**), RKO cell line (**D**). 5-FU—5-fluorouracil; EB5—28-propynoylbetulin; ECH147—29-diethoxyphosphoryl-28-propynoylbetulin; *—*p* < 0.05; **—*p* < 0.01; ***—*p* < 0.001.

The most substantial effects of compound exposure on *IL-8* gene expression were observed after 24 h. While CCD-841CoN and HT-29 cells exhibited the highest *IL-8* expression following betulin treatment, the SW1116 and RKO cell lines showed a distinct response, with cisplatin triggering the strongest induction. In normal colon epithelial cells, a significant increase in *IL-8* expression was detected after 24 h of exposure to betulin, compared to its derivatives (*p* < 0.001), 5-FU (*p* < 0.01), and the untreated cells (*p* < 0.001). A statistically significant downregulation was observed after treatment with ECH147, compared to cisplatin (*p* < 0.05), whereas EB5 led to an increase, compared to the control (*p* < 0.001) (Figure 3A).

In SW1116 cells (Figure 3B), the highest *IL-8* upregulation was observed after cisplatin treatment, relative to the control (*p* < 0.01), EB5, and ECH147 (both *p* < 0.001). Betulin also induced *IL-8* expression significantly compared to EB5 (*p* < 0.01) and ECH147 (*p* < 0.001). Conversely, ECH147 resulted in downregulation relative to 5-FU (*p* < 0.05).

HT-29 cells responded similarly to CCD-841CoN. Betulin induced the most substantial *IL-8* expression compared to EB5 and the control (*p* < 0.001), as well as compared to ECH147 (*p* < 0.01). The reference compounds also increased expression relative to untreated cells, with significance noted for 5-FU (*p* < 0.001) and cisplatin (*p* < 0.01). Additionally, 5-FU induced a higher expression of *IL-8* compared to EB5 (*p* < 0.05) (Figure 3C).

In contrast, the RKO cell line displayed a distinct expression pattern. Cisplatin led to a robust and statistically significant increase in *IL-8* expression compared to the control and all betulin derivatives (*p* < 0.001). Interestingly, 5-FU elicited a similar response, significantly upregulating *IL-8* compared to EB5 (*p* < 0.05), ECH147 (*p* < 0.001), and the control cells (*p* < 0.001) (Figure 3D).

### 2.2. Impact of Exposure Time to Investigated Compounds on IL-8 Gene Expression

**Figure 4:**
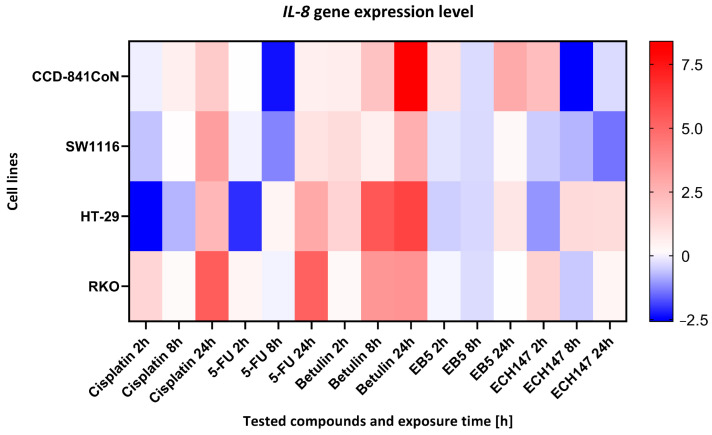
Heatmap illustrating the profile of *IL-8* gene expression changes in the tested cell lines at different exposure times to the compounds. The scale represents log2FC. 5-FU—5-fluorouracil; EB5—28-propynoylbetulin; ECH147—29-diethoxyphosphoryl-28-propynoylbetulin; Red—indicates an increase in expression level compared to the control; Blue—indicates a decrease in expression level compared to the control.

To investigate the temporal dynamics of *IL-8* gene regulation in response to compound exposure, a heatmap analysis was performed (Figure 4). *IL-8* mRNA transcript levels were assessed at 2, 8, and 24 h post-treatment in three CRC cell lines (HT-29, SW1116, RKO) and the non-cancerous colonocyte cell line, CCD-841CoN. Expression data are presented as log2 fold changes (log2FC) relative to the untreated controls. To compare differential responses between the cell lines, a heatmap was generated to visualize the relative *IL-8* expression patterns across time points and treatments.

Across all cell lines, a consistent pattern was observed: betulin elicited the most pronounced upregulation of *IL-8* expression, with maximal induction occurring at 24 h. In contrast, exposure to standard chemotherapeutic agents (cisplatin and 5-FU) resulted in downregulation or only modest changes in *IL-8* levels, especially after 24 h of treatment. The betulin derivatives EB5 and ECH147 exhibited variable effects depending on both the duration of exposure and the cellular context.

In normal colonocytes (CCD-841CoN), betulin significantly elevated *IL-8* expression after 24 h, whereas treatment with ECH147 and 5-FU led to a marked decrease. A similar response was observed in the HT-29 and SW1116 cancer cell lines, in which betulin induced robust *IL-8* upregulation compared to its derivatives and reference compounds. Notably, RKO cells responded to cisplatin with the strongest *IL-8* induction among all treatments, highlighting cell line-specific regulatory mechanisms. Among the compounds tested, the ECH147 derivative demonstrated the most favorable profile in terms of reducing *IL-8* expression. Given that IL-8 is a pro-inflammatory chemokine that is implicated in tumor progression, angiogenesis, and the formation of a protumorigenic microenvironment in CRC, the suppressive effect of ECH147 on *IL-8* expression suggests promising therapeutic potential for modulating the tumor-associated inflammatory response [11].

### 2.3. Time-Dependent Changes in IL-8 Protein Concentration in Cell Culture Medium in Colorectal Cancer Cell Lines

To validate the changes observed at the mRNA level, protein-level analyses were conducted using culture supernatants from colorectal cancer cell lines. Variations in cellular response were observed depending on the cell line, compound used, and exposure time.

**Figure 5:**
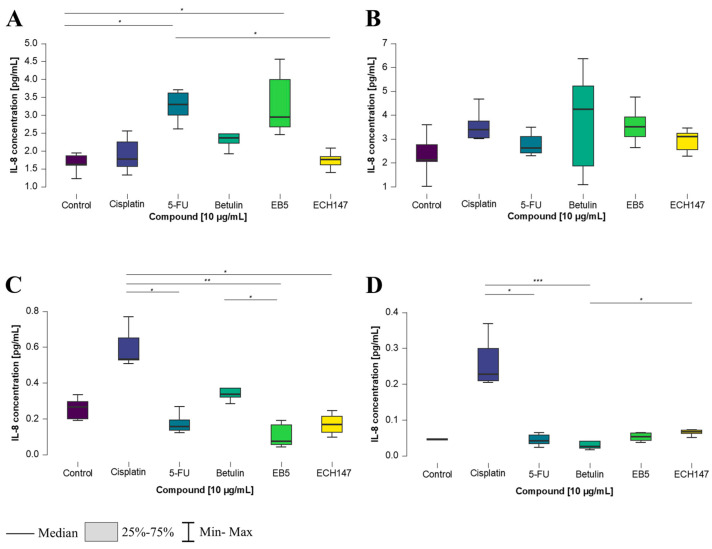
Changes in IL-8 protein concentration in cell culture medium in colorectal cancer cell lines after 2 h of exposure to the tested compounds: CCD-841CoN (**A**), SW1116 (**B**), HT-29 (**C**), RKO cell line (**D**). 5-FU—5-fluorouracil; EB5—28-propynoylbetulin; ECH147—29-diethoxyphosphoryl-28-propynoylbetulin; *—*p* < 0.05; **—*p* < 0.01; ***—*p* < 0.001.

In the CCD-841CoN cell line, a statistically significant increase in IL-8 concentration in the cell culture medium was detected following treatment with 5-FU, compared to untreated cells and those treated with the ECH147 derivative (*p* < 0.05). An elevated IL-8 level was also detected after exposure to the EB5 derivative, in comparison to control cells (*p* < 0.05) (Figure 5A). In the SW1116 cell line, no statistically significant changes in IL-8 levels were observed following 2 h of exposure to the tested compounds (Figure 5B).

In the HT-29 and RKO cell lines, increased IL-8 levels were detected following treatment with cisplatin. In HT-29 cells (Figure 5C), IL-8 concentration was significantly higher after cisplatin treatment in comparison to 5-FU (*p* < 0.05), as well as in comparison to the EB5 and ECH147 derivatives (*p* < 0.01 and *p* < 0.05, respectively). A reduction in IL-8 secretion was observed following EB5 exposure, compared to the unmodified compound, betulin (*p* < 0.05). In RKO cells, treatment with cisplatin resulted in significantly elevated IL-8 levels compared to 5-FU and betulin (*p* < 0.05 and *p* < 0.001, respectively). Furthermore, a decrease in IL-8 concentration was noted after treatment with betulin in comparison to the ECH147 derivative (*p* < 0.05) (Figure 5D).

**Figure 6:**
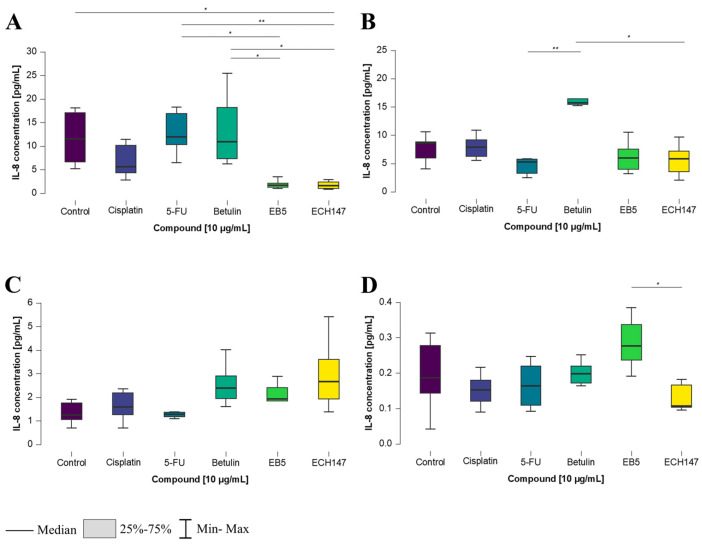
Changes in IL-8 protein concentration in cell culture medium in colorectal cancer cell lines after 8 h of exposure to the tested compounds: CCD-841CoN (**A**), SW1116 (**B**), HT-29 (**C**), RKO cell line (**D**). 5-FU—5-fluorouracil; EB5—28-propynoylbetulin; ECH147—29-diethoxyphosphoryl-28-propynoylbetulin; *—*p* < 0.05; **—*p* < 0.01.

An increase in IL-8 concentration was observed 8 h after treatment with the tested compounds. In normal colon epithelial cells, a statistically significant decrease in IL-8 levels was detected following treatment with the ECH147 derivative, compared to the control cells (*p* < 0.05), as well as in comparison to betulin and 5-FU (*p* < 0.05 and *p* < 0.01, respectively). Interestingly, a significant increase in IL-8 levels was also noted after treatment with betulin, compared to the EB5 derivative (*p* < 0.05) (Figure 6A).

In the SW1116 colorectal cancer cell line (Figure 6B), a significant increase in IL-8 concentration was observed after treatment with betulin, compared to both 5-FU and ECH147 (*p* < 0.01 and *p* < 0.05, respectively). In the HT-29 cell line, no significant differences in IL-8 levels were found between the tested compounds (Figure 6C).

In contrast, in the RKO cell line, protein levels were significantly higher after treatment with the EB5 derivative, compared to the ECH147 derivative (*p* < 0.05) (Figure 6D).

**Figure 7:**
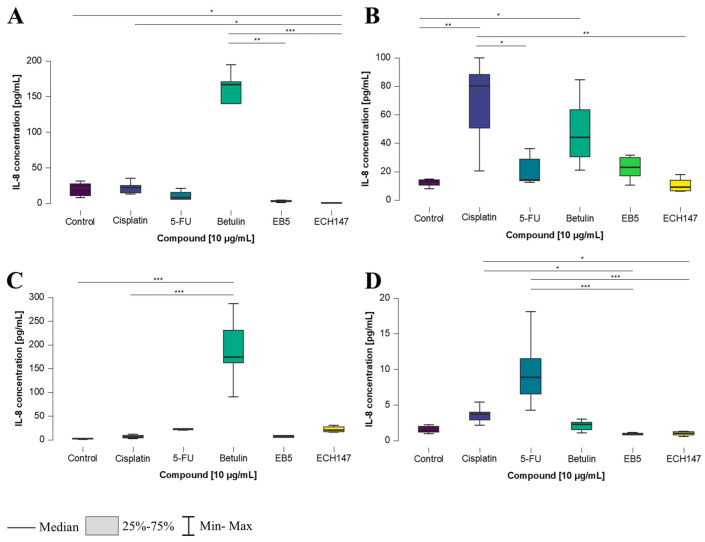
Changes in IL-8 protein concentration in cell culture medium in colorectal cancer cell lines after 24 h of exposure to the tested compounds: CCD-841CoN (**A**), SW1116 (**B**), HT-29 (**C**), RKO cell line (**D**). 5-FU—5-fluorouracil; EB5—28-propynoylbetulin; ECH147—29-diethoxyphosphoryl-28-propynoylbetulin; *—*p* < 0.05; **—*p* < 0.01; ***—*p* < 0.001.

IL-8 concentration levels showed the greatest variation after 24 h of treatment with the investigated compounds. In CCD-841CoN cells, a statistically significant increase in IL-8 secretion into the cell culture medium was detected following treatment with betulin, compared to its derivatives, EB5 and ECH147 (*p* < 0.01 and *p* < 0.001, respectively). Additionally, a significant decrease in IL-8 levels was noted after treatment with ECH147, compared to both control cells and those treated with cisplatin (*p* < 0.05) (Figure 7A). In the SW1116 cell line, a different response was observed. The highest IL-8 concentration was recorded following cisplatin treatment, with statistically significant differences compared to 5-FU (*p* < 0.05), as well as compared to untreated cells and ECH147 (*p* < 0.01). Moreover, treatment with betulin led to a significant increase in IL-8 protein secretion compared to the control (p < 0.05) (Figure 7B).

In HT-29 cells (Figure 7C), a significant response was observed after treatment with betulin, which increased IL-8 levels compared to both the control and cisplatin-treated cells (*p* < 0.001). In contrast, the RKO cell line responded differently (Figure 7D). The highest and most statistically significant increase in IL-8 concentration was observed following treatment with 5-FU, in comparison to all derivatives (*p* < 0.001). Interestingly, a significant decrease in IL-8 levels was also noted after treatment with the EB5 and ECH147 derivatives, compared to cisplatin (*p* < 0.05).

### 2.4. Time-Dependent Modulation of IL-8 Protein Levels by Investigated Compounds

**Figure 8:**
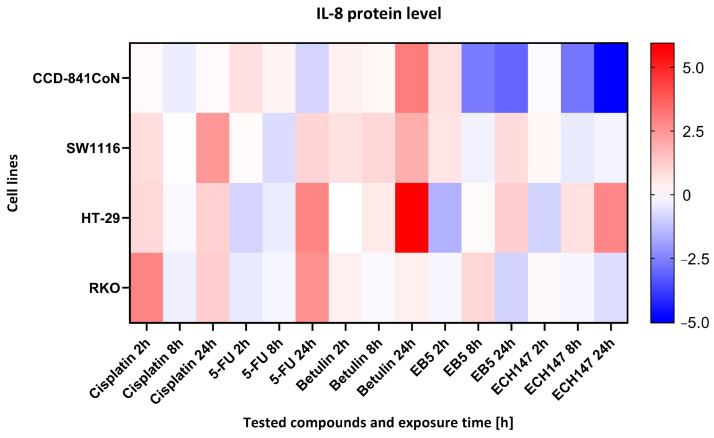
Heatmap illustrating the profile of IL-8 protein concentration changes in the cell culture medium of the tested cell lines at different exposure times to the compounds. The scale represents log2FC. 5-FU—5-fluorouracil; EB5—28-propynoylbetulin; ECH147—29-diethoxyphosphoryl-28-propynoylbetulin; Red—indicates an increase in expression level compared to the control; Blue—indicates a decrease in expression level compared to the control.

A heatmap was generated to visualize the relative levels of IL-8 protein secretion across different colon epithelial and colorectal cancer cell lines after treatment with selected compounds to highlight within-line differences in response (Figure 8).

In CCD-841CoN cells, the highest level of IL-8 secretion was observed following treatment with betulin, as indicated by the most intense red coloration on the heatmap. In contrast, the derivatives EB5 and ECH147 induced significantly lower IL-8 levels, which may suggest their potential anti-inflammatory activity in normal colon epithelial cells. In the SW1116 cell line, elevated IL-8 secretion was detected after treatment with both cisplatin and betulin. Notably, exposure to the derivatives EB5 and ECH147 resulted in decreased IL-8 levels, indicating a differential inflammatory response in this cancer cell line.

HT-29 cells exhibited a moderate increase in IL-8 levels, with the highest expression observed after 24 h of betulin treatment. Interestingly, in contrast to other cell lines, treatment with ECH147 in HT-29 cells also led to an increase in IL-8 secretion. The RKO cell line demonstrated a distinct response profile—IL-8 levels were most elevated after cisplatin treatment, whereas exposure to EB5 and ECH147 significantly reduced IL-8 secretion.

### 2.5. Molecular Docking

To determine the potential mechanism of action of the tested compounds, molecular docking was performed for all substances, except for cisplatin, whose specific chemical structure precludes the use of this method. This technique allows for the analysis of possible interactions with selected proteins, providing an alternative to costly and time-consuming experimental studies. The obtained results may contribute to the further structural modification of compounds to increase their biological efficacy. The crystal structure of IL-8 was downloaded from the Protein Data Bank database (PDB ID: 4XDX) (https://www.rcsb.org/, accessed on 28 April 2025). The tested substances were classified according to the value of the binding energy to the protein (Table 1), with a lower value of ΔG indicating a stronger affinity to the protein. The most favorable parameters were obtained for the EB5 derivative, whose binding energy was lower than that of the ECH147 derivative, betulin, and 5-FU.

**Figure 9:**
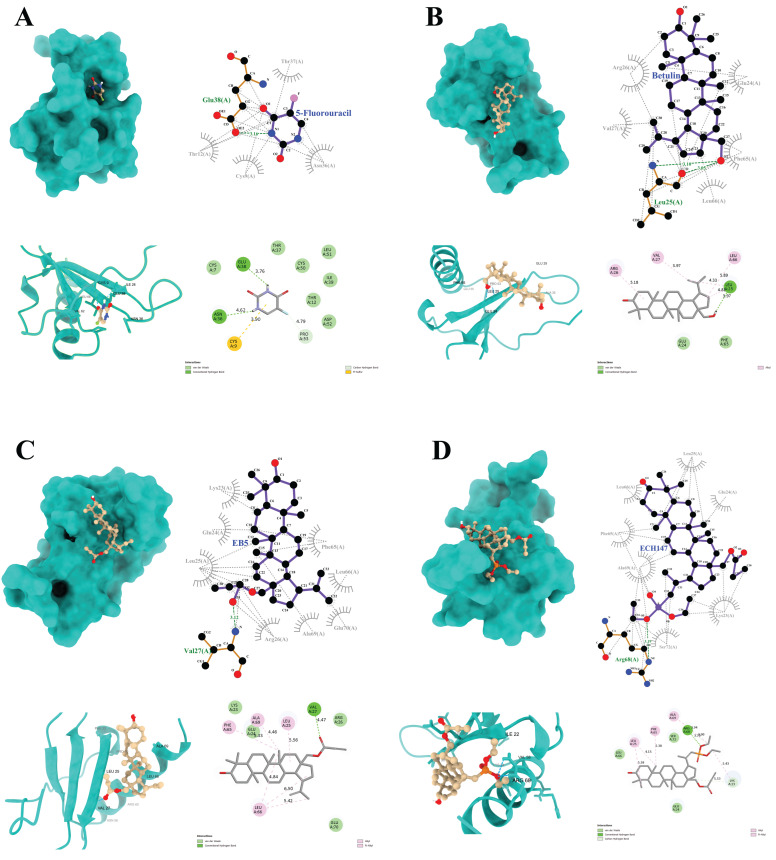
Graphical depiction of the interaction of IL-8 with the tested compounds: 5-fluorouracil (**A**), betulin (**B**), EB5 derivative (**C**), ECH147 derivative (**D**); green color indicates hydrogen bonds.

In order to further understand the interaction model, a binding analysis of the protein–ligand complex was performed (Figure 9).

The compound 5-FU forms two hydrogen bonds with the amino acid residues (Glu38 and Asn36) of the target protein. In addition, a π–sulfur interaction is observed at the Cys9 position, along with a C–H bond that involves the Pro53 residue (Figure 9A).

Betulin forms a single hydrogen bond with the Leu25 residue, as well as three alkyl interactions with Arg26, Val27, and Leu66 (Figure 9B). The EB5 derivative forms a complex with the IL-8 protein, which is primarily characterized by alkyl interactions with residues Phe65, Leu66, Ala69, and Leu25. Moreover, a strong hydrogen bond is formed at the Val27 position (Figure 9C).

A similar hydrogen bond is observed for the ECH147 derivative, although in this case, it occurs at the Arg68 position. In the ECH147–IL-8 complex, additional C–H interactions are noted at Lys23, along with alkyl interactions that involve Leu25, Phe65, and Ala69—residues that are also involved in the EB5 complex (Figure 9D).

Furthermore, all ligands exhibit numerous hydrophobic interactions with various residues of the protein, which may contribute to the overall stability of the complexes and enhance binding affinity to the molecular target.

## 3. Discussion

Investigating the underlying mechanisms and developing effective therapeutic strategies for CRC remains a persistent and complex challenge in biomedical research. CRC is the third most commonly diagnosed cancer worldwide and the second leading cause of cancer-related deaths. It occurs more frequently in men than in women [1]. The choice of treatment largely depends on the stage of the disease. In stages I and II, surgical resection of the tumor is typically the primary approach. In more advanced stages, treatment often includes not only surgery but also chemotherapy, immunotherapy, or targeted therapy [7]. However, these therapies remain far from ideal due to their toxic effects on the body and the growing problem of chemoresistance [1,5]. The use of plant-derived compounds has shown promising results in cancer therapy, offering benefits such as slowing tumor progression and reducing the side effects associated with conventional treatments. These compounds may inhibit cancer cell proliferation by disrupting cell cycle checkpoints and promoting programmed cell death [4]. One such compound is betulin, a naturally occurring substance found in significant quantities in grasses and trees. Due to its anticancer, antioxidant, and antibacterial properties, betulin is considered a promising candidate in cancer treatment research. Furthermore, research indicates that betulin has low toxicity toward normal, non-cancerous cells. Its chemical structure allows for straightforward modification, facilitating the development of new derivatives with enhanced pharmacokinetic profiles [12,13,14]. It has been shown that the hydroxyl groups (-OH) located at the C-3 and C-28 positions of betulin play a crucial role—their position, spacing, and orientation relative to the molecule’s ring system contribute to binding with active sites on the cell surface, which increases its biological activity [12,13,14,15]. Consequently, altering these functional groups can improve binding strength and boost the biological effectiveness of the molecule, leading to more potent and selective anticancer agents that target tumor cells [15]. Nonetheless, its application is limited by factors such as poor solubility, high hydrophobicity, and low bioavailability, which have driven scientists to explore potentially more effective derivatives [8].

The presence of inflammation is inherent in cancerous diseases. At the initial stage, it presents as sudden inflammation associated with tissue damage, which, after some time, becomes chronic. In this process, immune cells undoubtedly play a role [16,17]. Macrophages, as the first cells, appear at the site of damage. As a result of the reaction, they secrete chemotactic factors that attract other cells to the site of injury [16,17]. Their main feature of crucial importance is their ability to secrete a group of proteins called cytokines. Among these, interleukins are the largest and most widespread group [16,17]. Due to their action of binding to appropriate receptors, they lead to the activation of signaling pathways, through which cancer cells acquire their characteristic features, i.e., the unlimited ability to divide and avoid apoptosis or the ability to undergo angiogenesis and metastasis [8,9,18]. A key role in this process is played by IL-8, which, in the case of CRC, changes its concentration depending on the stage of the neoplasm [9,18].

The aim of this study was to assess the time-dependent effects of betulin and its novel derivatives, EB5 and ECH147, on IL-8 expression at both the gene and protein levels in human colorectal cancer cell lines at different clinical stages. Specifically, this study focused on the SW1116, HT-29, and RKO cell lines, which represent varying degrees of malignancy, from least to most aggressive [19]. Additionally, normal colon epithelial cells were included in the experiments. A secondary objective was to identify the most promising compound for a specific CRC cell line that may influence IL-8–associated signaling pathways. Based on previous MTT (3-[4,5-dimethylthiazol-2-yl]-2,5-diphenyltetrazolium bromide) assay results, a preselected concentration of 10 µg/mL was used for all tested compounds in this study [20].

To evaluate changes in *IL-8* gene expression in the studied cells, a real-time RT-qPCR analysis was performed. Significant alterations in mRNA expression were observed depending on the cell line, the compound tested, and the duration of exposure. *IL-8* gene expression was detected in all examined cell lines. Two compounds—betulin and its derivative, ECH147—elicited the most pronounced upregulation of *IL-8* expression 2 h after treatment. Interestingly, baseline *IL-8* mRNA levels were significantly higher in *BRAF*-mutant cell lines (HT-29 and RKO) compared to the *BRAF*-wild-type (*BRAF*-wt) CRC cell line, SW1116 [19]. After extended exposure (8 h), betulin treatment resulted in a substantial increase in *IL-8* expression across all cell lines, including normal colon epithelial cells, suggesting a potential immunomodulatory effect of betulin. Conversely, ECH147 led to a decrease in *IL-8* expression in the RKO cell line at the 8 h time point. Following 24 h of exposure, betulin continued to induce elevated *IL-8* mRNA levels in three tested cell lines (CCD-841CoN, SW1116, and HT-29). Notably, in the cell line with the highest malignancy grade, betulin caused a more pronounced downregulation of *IL-8* expression compared to conventional therapeutic agents. Furthermore, derivatives EB5 and ECH147 led to reduced IL-8 expression in HT-29 and RKO cell lines.

Similar findings were reported by Conciatori et al. [21], who analyzed the correlation between *BRAF* mutations and IL-8 expression across 28 CRC cell lines. Using gene expression profiling, next-generation sequencing (NGS), and protein analysis methods, such as Western blotting and immunofluorescence, they demonstrated that *BRAF* mutations and phosphatase and tensin homolog (PTEN) loss are associated with increased IL-8 levels in CRC models [21]. They further suggested that the BRAF/MEK/ERK signaling pathway plays a key role in IL-8 synthesis regulation, depending on genetic context, challenging the previously held view that PI3K/mTOR is the primary pathway involved. Additionally, they proposed that the BRAF/ERK2/CHOP axis modulates *IL-8* transcription by influencing C/EBP homologous protein (CHOP) localization and its response to specific inhibitors [21]. Their findings also indicated that the *BRAF* inhibitor dabrafenib exhibits differential effects, depending on the *BRAF* mutation status. In *BRAF^V600E^*-mutant cells, dabrafenib effectively blocks downstream signaling, thereby inhibiting extracellular signal-regulated kinase (ERK) and MAPK pathway activation and reducing IL-8 levels [21]. In contrast, in *BRAF* wild-type cells, this inhibitory effect is not observed or may even be reversed [21]. Our findings reflect a similar trend. The prolonged exposure (up to 24 h) of the SW1116, HT-29, and RKO cell lines led to variable changes in *IL-8* mRNA expression, depending on the cell line, and thus the *BRAF* mutation status. In BRAF^V600E^-mutant cell lines (HT-29 and RKO), treatment with the derivatives ECH147 and EB5 resulted in decreased *IL-8* expression after 8 h. However, this effect was transient, as an increase in *IL-8* expression was observed again after 24 h. These observations suggest that these derivatives may have the potential to modulate BRAF/ERK2/CHOP-related pathways, although further investigations are necessary to validate this hypothesis.

In a study conducted by Kaleta-Richter et al. [22], the authors investigated the effects of combination therapy using hypericin and photodynamic therapy (PDT) on the secretion of both IL-8 and IL-10 in CRC cell lines. Using cytotoxicity assays, flow cytometry-based methods, and Bio-Plex protein-level analysis, they observed findings consistent with ours [22]. They reported that baseline IL-8 secretion varies depending on the clinical stage of CRC. Specifically, they showed that cell lines derived from tumors of lower clinical grade (e.g., SW480) secreted higher levels of IL-8 compared to those from higher-grade tumors (e.g., SW620) [22]. The authors suggested that this elevated IL-8 production may stimulate chemotaxis and promote compensatory mechanisms in more aggressive cancer cells. We observed a similar pattern in our experiments [22]. SW1116 cells consistently secreted significantly more IL-8 protein into the culture medium than lower-grade tumor cell lines, regardless of the incubation time. Moreover, the highest increase in IL-8 concentration in the medium was observed after betulin treatment in the SW1116 cell line. This may be associated with the absence of *BRAF* mutations in these cells, as previously discussed. Interestingly, the more aggressive cell lines, HT-29 and RKO, responded differently. A decrease in IL-8 secretion into the medium was observed following treatment with the two derivatives, EB5 and ECH147. These protein-level findings are consistent with the gene expression data, further supporting the notion that the interleukin response in CRC cells is strongly influenced by the *BRAF^V600E^* mutation status.

In another study conducted by Lubczyńska et al. [23], the effects of betulin and its acyl derivatives on the gene expression profile associated with inflammation were evaluated using the colorectal cancer cell line HT-29 and normal human dermal fibroblasts (NHDF). To this end, the authors employed HG-U133A oligonucleotide microarray technology to visualize changes in gene expression [23]. This study demonstrated that both betulin and its acyl derivatives modulate genes involved in inflammatory responses, including those associated with the cytokine-mediated signaling pathway (e.g., *CHUK*, *CXCL9*) [23].

Our findings further support the observation that the pentacyclic triterpene betulin modulates the expression of pro-inflammatory cytokines. Moreover, we observed that structural modifications of the betulin molecule yielded distinct effects depending on the cell line used and the type of modification applied. In our study, betulin consistently increased IL-8 expression at all examined time points, suggesting its potential role in activating IL-8-associated signaling pathways, leading to a transient upregulation of its expression.

In a study conducted by Malicki et al. [24], the effects of simvastatin on the secretion levels of two pro-inflammatory cytokines, IL-6 and IL-8, were assessed in CRC tumor tissue and adjacent healthy margins, as well as in vitro using the HT-29 and Caco-2 cell lines [24]. Using real-time RT-qPCR and enzyme-linked immunosorbent assay (ELISA), the authors demonstrated that elevated mRNA and protein expression levels of IL-8 correlated with the clinical stage of CRC [24]. Furthermore, their findings confirmed that the surgical resection of the tumor resulted in an immediate decrease in serum CXCL8 levels, highlighting its potential as a biomarker for tumor burden. In addition, in vitro experiments using simvastatin showed a significant reduction in IL-6 and IL-8 levels, suggesting that simvastatin may exert anti-inflammatory effects that could be relevant to CRC progression [24]. The authors concluded that CRC carcinogenesis is accompanied by the increased synthesis and secretion of IL-6 and IL-8, underscoring the need to identify natural compounds capable of downregulating the expression of these pro-inflammatory cytokines [24].

In our study, we observed a reduction in both the expression and secretion of IL-8 protein, particularly in response to specific betulin derivatives such as EB5 and ECH147. These results suggest that selected structural modifications of betulin may exert an inhibitory effect on CRC-related inflammation, potentially by attenuating IL-8-driven signaling pathways involved in tumor progression. However, further research is required to confirm this hypothesis.

Riekstina et al. [25] also conducted a noteworthy study in which they developed colloidal formulations of betulin and investigated their effects on cell viability, proliferation, and cytokine release in the NHDF cell line. To assess the influence of betulin on IL-8 secretion, they utilized fluorescence microscopy and quantified protein levels using ELISA. Their findings revealed that betulin significantly increased IL-8 secretion in normal fibroblasts [25]. This observation aligns with our own results, in which a similar upregulation of IL-8 secretion was noted in the normal human colon epithelial cell line, CCD-841CoN, following treatment with betulin. However, the study by Riekstina et al. [25] did not explore the underlying molecular mechanisms responsible for this effect. Therefore, additional research is necessary to clarify how betulin modulates IL-8 expression in non-cancerous cells. A better understanding of this phenomenon could help determine whether such an effect represents a temporary activation of immune signaling or indicates a broader, potentially adverse pro-inflammatory response in normal tissues.

To further investigate the potential mechanism of action of the studied compounds, we performed molecular docking analyses targeting the CXCL8 protein. A detailed comparison of docking parameters for each compound is presented in Table 2.

A similar approach was reported by Alshahrani et al. [26], who used in silico methods to evaluate the binding affinity and molecular dynamics of selected natural compounds—isohydnocarpin, chitranone, and 1-hydroxyrutaecarpine—all of which have recognized anticancer potential [26]. Their results revealed strong binding interactions with CXCL8, with calculated binding energies ranging from −9.9 to −9.1 kcal/mol, supported by the formation of multiple hydrogen bonds [26].

In our study, we found that betulin, a pentacyclic triterpene, also binds to CXCL8 with notable affinity (−8.0 kcal/mol). Moreover, structural modifications of betulin had a measurable impact on binding strength. The derivative EB5, which features propynoyl group substitutions, showed improved binding energy (−8.7 kcal/mol), while ECH147 displayed a similar affinity to the unmodified compound. These results indicate that betulin derivatives, particularly EB5, may have the capacity to interact directly with CXCL8, potentially modulating its biological activity.

Although EB5 exhibits a higher binding energy to IL-8 in docking studies, ECH147 shows stronger functional inhibition in vitro. This may be due to differences in interaction profiles, such as variations in hydrogen bonding, hydrophobic, and van der Waals interactions, which together could influence biological activity beyond binding affinity alone. In addition, the EB5 derivative possesses a modification at the C-28 carbon position, which studies have shown to be associated with binding strength to cellular proteins [15]. This apparent discrepancy may stem from the fact that docking only evaluates the theoretical binding potential under static conditions, while biological activity is influenced by multiple factors, including cell permeability, metabolic stability, and the specific location of ligand interaction within the protein structure. It is also possible that ECH147, despite its slightly lower binding energy, binds to a more functionally critical site on IL-8, leading to a more pronounced biological effect. While these findings are promising, they remain preliminary and require further validation through pharmacokinetic and functional studies to determine whether these interactions translate into meaningful biological or therapeutic effects.

In summary, our findings indicate that betulin derivatives can reduce IL-8 expression in colorectal cancer cell lines with distinct genetic and epigenetic backgrounds. Notably, the mechanism of action of the derivatives EB5 and ECH147 may be associated with the presence of a *BRAF* mutation, as the most pronounced effects were observed in cell lines that harbor this mutation, in contrast to the SW1116 line, which is a BRAF wild-type. Furthermore, we confirmed that less aggressive (lower-grade) cancer cell lines exhibited higher basal levels of IL-8 expression compared to more advanced tumor lines. Our time-course experiments suggest that the effects of both betulin and its derivatives are transient, with peak activity observed around 8 h post-exposure.

Interestingly, unmodified betulin significantly increased IL-8 expression, which is a cytokine that is known to promote angiogenesis and metastasis in cancer, underscoring the importance of structural modifications in modulating its biological effects. Our in silico molecular docking results support the experimental data, revealing that EB5 and ECH147 show the strongest binding affinities to CXCL8, which may explain their inhibitory impact on IL-8 expression. This study also demonstrated a time-dependent effect of betulin and its derivative, which may be related to the stability of their binding to proteins in the culture medium or a transient influence of betulin and similar derivatives on *IL-8* transcription. In clinical practice, this implies that treatment schedules need to be carefully designed to ensure sustained effective exposure, thereby optimizing therapeutic benefits while reducing the risk of toxicity. These findings highlight the therapeutic promise of these compounds as modulators of inflammation in cancer. Future studies should focus on validating these effects in in vivo systems and exploring the integration of these compounds into advanced drug delivery platforms—such as nanoparticles or polymer-based carriers—to enhance their selectivity, bioavailability, and clinical applicability [27,28,29].

## 4. Materials and Methods

### 4.1. In Vitro Culture of Colorectal Cancer Cell Lines and Normal Colonocytes

The colorectal cancer cell lines, RKO, HT-29, and SW1116 (CRL-2577, HTB-38, CCL-233; ATCC, Manassas, VA, USA), and the normal CCD-841CoN cell line (CRL-1790, ATCC, Manassas, VA, USA) were routinely cultured at 37 °C in a 5% CO_2_ incubator (Direct Heat CO_2_; Thermo Scientific, Waltham, MA, USA). The cell lines were maintained in media specific to each type, in accordance with the manufacturer’s recommendations. RKO and CCD-841CoN cells were cultured in Eagle’s minimum essential medium (EMEM), while HT-29 cells were cultured in McCoy’s 5A medium. SW1116 cells were grown in Leibovitz’s L-15 medium. All culture media were supplemented with 10% fetal bovine serum (EuroClone, Milan, Italy), and 50 mg/mL of gentamicin (BioWhittaker, Lonza, Basel, Switzerland) was added to prevent microbial contamination.

### 4.2. Cell Culture Treatment and Time Exposure

Betulin derivatives EB5 and ECH147 were obtained from the Department of Organic Chemistry, Faculty of Pharmaceutical Sciences in Sosnowiec, Medical University of Silesia in Katowice. The synthesis and detailed characterization of EB5 have been documented by Boryczka et al. [13], whereas the preparation of ECH147 was described by Chrobak et al. [30]. To evaluate the effect of betulin and its derivatives on IL-8 expression levels, cells were treated with the test compounds betulin, EB5, ECH147, 5-fluorouracil, and cisplatin at a concentration of 10 µg/mL. Cisplatin and 5-FU were used as reference compounds due to their established roles as standard chemotherapeutic agents in colorectal cancer treatment. The concentration was selected based on previous studies from the cytotoxicity assay [20]. In addition, to evaluate the effect of the time of exposure of the cells to the compounds, they were kept sequentially for 2, 8, and 24 h. The control cells were not treated with compounds. Three biological replicates were performed. After this time, the culture medium was collected and centrifuged, and the supernatant was preserved for protein-level analyses. Once the culture medium was removed, 500 µL of TRIzol reagent (Sigma-Aldrich, St Louis, MO, USA) per well was added to the cells to protect the RNA for further molecular-level studies.

### 4.3. Ribonucleic Acid Extraction and the Qualitative and Quantitative Evaluation of Extracts

Total RNA was extracted from treated cells using the TRIzol reagent (Sigma-Aldrich, St Louis, MO, USA) in accordance with the manufacturer’s guidelines. The purity and concentration of the obtained RNA were determined via agarose gel electrophoresis and spectrophotometric analysis with the MaestroNano MN-913 (MaestroGen Inc., Las Vegas, NV, USA). The extracts were then used as the basis for evaluating gene expression changes at the mRNA level.

### 4.4. Gene-Level Alteration Assessments via RT-qPCR in Real Time

The expression of the *IL-8* gene was analyzed using real-time RT-qPCR on the LightCycler^®^ 480 System (Roche, Basel, Switzerland). The GoTaq^®^ 1-Step RT-qPCR System (Promega, Madison, WI, USA) was used, following the same reaction conditions described in a previous study by Kruszniewska-Rajs et al. [31]. The following primer sequences were used for *IL-8* gene expression analysis: forward, 5′-CTCTAACTCTTTATATAGGAAGT-3′; reverse, 5′-AGTAGCTGGCAGAGCTGTG-3′. β-actin (*ACTB*) served as the reference gene. The relative mRNA expression levels were calculated using the 2^−ΔΔCt^ method. Each sample was analyzed in triplicate. Reaction specificity was verified through a melting curve analysis and via electrophoresis of the amplification products on a 2% agarose gel.

### 4.5. Evaluation of IL-8 Protein Change in the Cell Culture Medium Using Proximity Ligation Assay Technology (PLA)

IL-8 protein levels in the cell culture supernatant were quantified using the commercially available Human IL-8 ProQuantum Immunoassay Kit (ThermoFisher Scientific, Waltham, MA, USA), in accordance with the manufacturer’s instructions. The method employs PLA technology, in which two antibodies specific to different epitopes of IL-8 bind to the target protein, facilitating the formation of a DNA-labeled complex. In the presence of IL-8, the proximity of the oligonucleotide-conjugated antibodies enables signal amplification via quantitative PCR (qPCR), resulting in a fluorescent signal that is proportional to the concentration of IL-8 in the sample. The test exhibits a sensitivity range from 0.5 to 10,000 pg/mL and a detection limit of 0.5 pg/mL. Samples were diluted 1:2 prior to analysis. The qPCR reaction was conducted on the QuantStudio 7 Pro Dx Real-Time PCR System (ThermoFisher Scientific Inc., Waltham, MA, USA), and the resulting data were analyzed using the ProQuantum online platform (apps.thermofisher.com/apps/proquantum (accessed on 29 April 2025).

### 4.6. Molecular Docking—In Silico Studies

Molecular docking was performed for 5-FU, betulin, EB5, and ECH147 derivatives to analyze their interactions with IL-8 (CXCL8) as the target protein. The in silico study aimed to elucidate the potential mechanism of action and determine the strength and specificity of the binding between the ligand and these proteins [32]. The structures of the compounds were obtained from the PubChem database (https://pubchem.ncbi.nlm.nih.gov/, accessed on 28 April 2025) in SDF format and subsequently converted to mol2 format using ChimeraX 1.9 software. Crystal models of IL-8 (PDB ID: 4XDX) were downloaded from the RCSB database (https://www.rcsb.org/, accessed on 28 April 2025) in PDB format.

Prior to molecular docking, the protein structures were properly prepared. Using ChimeraX 1.9, the original solvents and other non-essential molecules were removed. Subsequently, using AutoDock Tools 1.5.7, water molecules were eliminated, hydrogen atoms were added, and electrostatic charges were calculated. The prepared structures were then saved in PDBQT format. Molecular docking was conducted using AutoDock Vina 1.1.2. To visualize the results, Discovery Studio 2025, ChimeraX 1.9, and LigPlot+ 2.2 software were employed.

### 4.7. Statistical Analysis

Statistical analyses were carried out using STATISTICA software (version 13.3; Tibco Inc., Palo Alto, CA, USA). All experimental setups were conducted in triplicate. Qualitative outcomes were illustrated using box-and-whisker plots, with visualization performed using JASP software version 0.19.1.0 (University of Amsterdam, Amsterdam, The Netherlands). GraphPad Prism 9.0.2 (GraphPad Software, San Diego, CA, USA) was used to generate heatmaps. Quantitative variables with non-normal distributions were expressed as medians, along with the corresponding interquartile ranges. The distribution of data was evaluated using the Shapiro–Wilk test. To compare differences between groups, the Kruskal–Wallis non-parametric ANOVA was employed, followed by a post hoc test based on mean rank comparisons. A *p*-value less than 0.05 was considered indicative of statistical significance.

## 5. Limitations and Future Directions

Betulin, a natural triterpenoid, is relatively well-studied for its anticancer, antiviral, and anti-inflammatory properties [33]. However, due to its poor bioavailability, it often undergoes chemical modifications to generate more active and soluble derivatives, which are increasingly being synthesized and investigated in preclinical studies. Additionally, betulin and its derivatives remain experimental compounds and have not yet been approved for clinical use in CRC treatment. In the context of colorectal cancer, the current literature only offers limited data on specific betulin derivatives such as EB5 and ECH147, particularly in relation to specific colorectal cancer cell lines and their impact on IL-8 expression.

A limitation of these studies is the predominant use of 2D in vitro cultures, which fail to accurately replicate the complexity of the in vivo tumor microenvironment [33,34]. To overcome this issue, future research should implement more advanced 3D culture systems, such as spheroids or organoids, which better mimic the physiological and architectural features of tumors. These models could provide more reliable and translational insights into the biological activity and therapeutic potential of betulin derivatives. Moreover, no in vivo studies using animal models have yet been conducted for EB5 or ECH147, emphasizing the strictly preclinical nature of the current findings. The present study primarily aims to assess the potential of these compounds for future translational and in vivo research.

In addition to IL-8, future investigations should also consider other key interleukins involved in the progression of colorectal cancer, such as IL-6 and IL-10, which hold significant clinical relevance. The scope of research could also be broadened through the application of advanced molecular techniques to more precisely identify the molecular targets of these compounds. One promising approach is Proteolysis-Targeting Chimera (PROTAC) technology, which utilizes the ubiquitin–proteasome system to selectively degrade disease-associated proteins, thereby reducing their levels rather than simply inhibiting their activity [35,36].

Furthermore, beyond protein-level investigations, the use of NGS could facilitate the identification of novel gene expression profiles that are altered in response to treatment with betulin derivatives. Combined with bioinformatics analyses, this strategy would help elucidate the mechanisms of action and identify potential biomarkers or therapeutic targets [37,38].

## 6. Conclusions

Our findings demonstrate that IL-8 expression levels vary significantly across different CRC cell lines, correlating with the malignancy grade and likely reflecting underlying genetic and epigenetic differences. Betulin and its synthetic derivatives, EB5 and ECH147, exhibit immunomodulatory properties, with ECH147 showing the most potent effect by reducing IL-8 expression in a time-dependent manner. Notably, these derivatives display higher affinity for the CXCL8 protein than conventional chemotherapeutics. This suggests that the incorporation of a propynoyl group may enhance molecular interaction with IL-8, contributing to improved bioactivity. These results highlight the potential of ECH147 as a promising lead compound for targeting inflammatory pathways in CRC and further preclinical investigation into its mechanism of action and therapeutic applicability. Moreover, our data suggest that the mechanism of action of the tested compounds may be closely linked to the *BRAF* mutation status of each CRC cell line, which could influence their response profiles. However, this hypothesis requires further exploration through detailed studies of the relevant molecular signaling pathways.

## Figures and Tables

**Table 1:** Binding affinities of 5-FU, betulin, EB5, and ECH147 in complexes with IL-8.

Compound	ΔG [kcal/mol]
EB5	−8.7
Betulin	−8.0
ECH147	−7.9
5-FU	−4.4

EB5—28-propynoylbetulin; ECH147—29-diethoxyphosphoryl-28-propynoylbetulin; 5-FU—5-fluorouracil.

**Table 2:** Comparison of docking parameters of EB5, betulin, ECH147, and 5-FU with the IL-8 protein.

Parameter	EB5	Betulin	ECH147	5-FU
Binding Energy [kcal/mol]	−8.7	−8.0	−7.9	−4.4
Hydrogen Bonds	1	1	1	2
Residues H-bonded	Val27	Leu25	Arg68	Glu38; Asn36
Hydrophobic Interactions	8	5	7	4
Carbon–Hydrogen Bonds	0	0	1	1
Van der Waals Interactions	4	2	3	7
Alkyl Interactions	4	3	3	0
Π–Sulfur Interactions	0	0	0	1

EB5—28-propynoylbetulin; ECH147—29-diethoxyphosphoryl-28-propynoylbetulin; 5-FU—5-fluorouracil.

## Data Availability

The raw data supporting the conclusions of this article will be made available by the authors on request.

## Abbreviations

The following abbreviations are used in this manuscript:

5-FU	5-fluorouracil
ACTB	β-actin
BRAF	V-raf murine sarcoma viral oncogene homolog B
BRAF-wt	V-raf murine sarcoma viral oncogene homolog B wild-type
CAFs	Cancer-associated fibroblasts
CHOP	C/EBP homologous protein
CRC	Colorectal cancer
CXCL8	C-X-C motif chemokine ligand 8
E	Glutamic acid
EB5	28-propynoylbetulin
ECH147	29-diethoxyphosphoryl-28-propynoylbetulin
EGFR	Epidermal growth factor receptor
ELISA	Enzyme-linked immunosorbent assay
EMEM	Eagle’s minimum essential medium
ERK	Extracellular signal-regulated kinase
GI	Gastrointestinal
IL-8	Interleukin-8
Log2FC	Log2 fold changes
MAPK	Mitogen-activated protein kinase
MMPs	Matrix metalloproteinases
MTT	3-[4,5-dimethylthiazol-2-yl]-2,5-diphenyltetrazolium bromide
NGS	Next-generation sequencing
NHDF	Normal human dermal fibroblasts
PDB	Protein Data Bank
PLA	Proximity ligation assay technology
PROTAC	Proteolysis-targeting chimera
PTEN	Phosphatase and tensin homolog
qPCR	Quantitative PCR
TAMs	Tumor-associated macrophages
V	Valine

## References

[1] Puzzo M., De Santo M., Morelli C., Leggio A., Catalano S., Pasqua L. (2025). Colorectal cancer: Current and future therapeutic approaches and related technologies addressing multidrug strategies against multiple level resistance mechanisms. Int. J. Mol. Sci..

[2] Tsukanov V.V., Vasyutin A.V., Tonkikh J.L. (2025). Risk factors, prevention and screening of colorectal cancer: A rising problem. World J. Gastroenterol..

[3] Fadlallah H., El Masri J., Fakhereddine H., Youssef J., Chemaly C., Doughan S., Abou-Kheir W. (2024). Colorectal cancer: Recent advances in management and treatment. World J. Clin. Oncol..

[4] Swain J., Preeti, Mohanty C., Bajoria A.A., Patnaik S., Ward Gahlawat A., Nikhil K., Mohapatra S.R. (2025). Deciphering the metabolic landscape of colorectal cancer through the lens of AhR-mediated intestinal inflammation. Discov. Oncol..

[5] Saoudi González N., Ros J., Baraibar I., Salvà F., Rodríguez-Castells M., Alcaraz A., García A., Tabernero J., Élez E. (2024). Cetuximab as a key partner in personalized targeted therapy for metastatic colorectal cancer. Cancers.

[6] Madej M., Gola J., Chrobak E. (2023). Synthesis, pharmacological properties, and potential molecular mechanisms of antitumor activity of betulin and its derivatives in gastrointestinal cancers. Pharmaceutics.

[7] Chrobak E., Świtalska M., Wietrzyk J., Bębenek E. (2025). New difunctional derivatives of betulin: Preparation, characterization and antiproliferative potential. Molecules.

[8] Meier C., Brieger A. (2025). The role of IL-8 in cancer development and its impact on immunotherapy resistance. Eur. J. Cancer..

[9] Bazzichetto C., Milella M., Zampiva I., Simionato F., Amoreo C.A., Buglioni S., Pacelli C., Le Pera L., Colombo T., Bria E. (2022). Interleukin-8 in colorectal cancer: A systematic review and meta-analysis of its potential role as a prognostic biomarker. Biomedicines.

[10] Fellhofer-Hofer J., Franz C., Vey J.A., Kahlert C., Kalkum E., Mehrabi A., Halama N., Probst P., Klupp F. (2024). Chemokines as prognostic factor in colorectal cancer patients: A systematic review and meta-analysis. Int. J. Mol. Sci..

[11] Li W., Chen F., Gao H., Xu Z., Zhou Y., Wang S., Lv Z., Zhang Y., Xu Z., Huo J. (2023). Cytokine concentration in peripheral blood of patients with colorectal cancer. Front. Immunol..

[12] Rzeski W., Stepulak A., Szymański M., Juszczak M., Grabarska A., Sifringer M., Kaczor J., Kandefer-Szerszeń M. (2009). Betulin elicits anti-cancer effects in tumour primary cultures and cell lines in vitro. Basic Clin. Pharmacol. Toxicol..

[13] Boryczka S., Bębenek E., Wietrzyk J., Kempińska K., Jastrzębska M., Kusz J., Nowak M. (2013). Synthesis, structure and cytotoxic activity of new acetylenic derivatives of betulin. Molecules.

[14] Król S.K., Kiełbus M., Rivero-Müller A., Stepulak A. (2015). Comprehensive review on betulin as a potent anticancer agent. Biomed. Res. Int..

[15] Cabaj J., Bąk W., Wróblewska-Łuczka P. (2021). Anti-cancer effect of betulin and its derivatives, with particular emphasis on the treatment of melanoma. J. Pre-Clin. Clin. Res..

[16] Wang X., Zhang J., Fang L., Tang X. (2025). Angel and devil: The protective immunity and pathogenic inflammation of tissue resident memory T cells in ulcerative colitis. Front. Immunol..

[17] Li Q., Geng S., Luo H., Wang W., Mo Y.Q., Luo Q., Wang L., Song G.B., Sheng J.P., Xu B. (2024). Signaling pathways involved in colorectal cancer: Pathogenesis and targeted therapy. Signal Transduct. Target Ther..

[18] Pecqueux M., Brückner F., Oehme F., Hempel S., Baenke F., Riediger C., Distler M., Weitz J., Kahlert C. (2024). Preoperative IL-8 levels as prognostic indicators of overall survival: An extended follow-up in a prospective cohort with colorectal liver metastases. BMC Cancer.

[19] Ahmed D., Eide P.W., Eilertsen I.A., Danielsen S.A., Eknæs A., Hektoen M., Lind G.E., Lothe R.A. (2013). Epigenetic and genetic features of 24 colon cancer cell lines. Oncogenesis.

[20] Madej M., Kruszniewska-Rajs C., Kimsa-Dudek M., Synowiec-Wojtarowicz A., Chrobak E., Bębenek E., Boryczka S., Głuszek S., Adamska J., Kubica S. (2024). The influence of betulin and its derivatives on selected colorectal cancer cell lines’ viability and their antioxidant systems. Cells.

[21] Conciatori F., Bazzichetto C., Amoreo C.A., Sperduti I., Donzelli S., Diodoro M.G., Buglioni S., Falcone I., Shirasawa S., Blandino G. (2020). BRAF status modulates Interelukin-8 expression through a CHOP-dependent mechanism in colorectal cancer. Commun. Biol..

[22] Kaleta-Richter M., Aebisher D., Jaworska D., Czuba Z., Cieślar G., Kawczyk-Krupka A. (2020). The influence of hypericin-mediated photodynamic therapy on interleukin-8 and -10 secretion in colon cancer cells. Integr. Cancer Ther..

[23] Lubczyńska A., Bębenek E., Garncarczyk A., Wcisło-Dziadecka D. (2023). Evaluation of the effect of betulin and its alkynyl derivatives on the profile of changes in gene expression of the inflammatory process of colorectal adenocarcinoma cells (HT-29 cell line). Processes.

[24] Malicki S., Winiarski M., Matlok M., Kostarczyk W., Guzdek A., Konturek P.C. (2009). IL-6 and IL-8 responses of colorectal cancer in vivo and in vitro cancer cells subjected to simvastatin. J. Physiol. Pharmacol..

[25] Riekstina U., Vitolina S., Goluba K., Jekabsons K., Muceniece R., Berzins R., Rizhikovs J., Godina D., Teresko A., Paze A. (2023). Effect of betulin colloidal particles on proliferation and cytokine secretion of human skin fibroblasts. Plants.

[26] Alshahrani M.Y., Alkhathami A.G., Almoyad M.A.A., Ahmad M.Z., Mohanto S., Ahmad W., Wahab S. (2025). Phytochemicals as potential inhibitors of interleukin-8 for anticancer therapy: In silico evaluation and molecular dynamics analysis. J. Biomol. Struct. Dyn..

[27] Olakowska E., Wlaszczuk A., Turek A., Borecka A., Liskiewicz A., Wawro D., Kasperczyk J., Jedrzejowska-Szypulka H. (2022). Effects of 17-β-estradiol released from shape-memory terpolymer rods on sciatic nerve regeneration after injury and repair with chitosan nerve conduit in female rats. J. Appl. Biomed..

[28] Rech J., Wilińska J., Turek A. (2020). Application of fibrin in drug technology: Achievements and perspectives. Postępy Hig. Med. Dośw..

[29] Uner B., Akyildiz E.O., Kolci K., Reis R. (2025). Nanoparticle formulations for intracellular delivery in colorectal cancer therapy. AAPS PharmSciTech.

[30] Chrobak E., Bębenek E., Kadela-Tomanek M., Latocha M., Jelsch C., Wenger E., Boryczka S. (2016). Betulin phosphonates; synthesis, structure, and cytotoxic activity. Molecules.

[31] Kruszniewska-Rajs C., Strzałka-Mrozik B., Kimsa-Dudek M., Synowiec-Wojtarowicz A., Chrobak E., Bębenek E., Boryczka S., Głuszek S., Gola J.M. (2022). The influence of betulin and its derivatives EB5 and ECH147 on the antioxidant status of human renal proximal tubule epithelial cells. Int. J. Mol. Sci..

[32] Martis E.A.F., Téletchéa S. (2025). Ten quick tips to perform meaningful and reproducible molecular docking calculations. PLoS Comput. Biol..

[33] Serrafi A., Wasilewski A. (2025). Synthesis and antimicrobial activity of new betulin derivatives. Sci. Rep..

[34] Tâlvan C.D., Budișan L., Tâlvan E.T., Grecu V., Zănoagă O., Mihalache C., Cristea V., Berindan-Neagoe I., Mohor C.I. (2024). Serum interleukins 8, 17, and 33 as potential biomarkers of colon cancer. Cancers.

[35] Yan S., Zhang G., Luo W., Xu M., Peng R., Du Z., Liu Y., Bai Z., Xiao X., Qin S. (2024). PROTAC technology: From drug development to probe technology for target deconvolution. Eur. J. Med. Chem..

[36] Qin S., Xiao X. (2025). Key advances and application prospects of PROTAC technologies in the next 5 years. Future Med. Chem..

[37] Wang H., Huang J., Fang X., Liu M., Fan X., Li Y. (2025). Advances in next-generation sequencing (NGS) applications in drug discovery and development. Expert Opin. Drug Discov..

[38] Niazi S.K., Mariam Z. (2025). Artificial intelligence in drug development: Reshaping the therapeutic landscape. Ther. Adv. Drug Saf..

